# Measuring inequalities in the distribution of health workers: the case of Tanzania

**DOI:** 10.1186/1478-4491-7-4

**Published:** 2009-01-21

**Authors:** Michael A Munga, Ottar Mæstad

**Affiliations:** 1 National Institute for Medical Research, Dar es Salaam, Tanzania; 2 Centre for International Health, University of Bergen, Bergen, Norway; 3 Chr Michelsen Institute, Bergen, Norway

## Abstract

**Background:**

The overall human resource shortages and the distributional inequalities in the health workforce in many developing countries are well acknowledged. However, little has been done to measure the degree of inequality systematically. Moreover, few attempts have been made to analyse the implications of using alternative measures of health care needs in the measurement of health workforce distributional inequalities. Most studies have implicitly relied on population levels as the only criterion for measuring health care needs. This paper attempts to achieve two objectives. First, it describes and measures health worker distributional inequalities in Tanzania on a per capita basis; second, it suggests and applies additional health care needs indicators in the measurement of distributional inequalities.

**Methods:**

We plotted Lorenz and concentration curves to illustrate graphically the distribution of the total health workforce and the cadre-specific (skill mix) distributions. Alternative indicators of health care needs were illustrated by concentration curves. Inequalities were measured by calculating Gini and concentration indices.

**Results:**

There are significant inequalities in the distribution of health workers per capita. Overall, the population quintile with the fewest health workers per capita accounts for only 8% of all health workers, while the quintile with the most health workers accounts for 46%. Inequality is perceptible across both urban and rural districts. Skill mix inequalities are also large. Districts with a small share of the health workforce (relative to their population levels have an even smaller share of highly trained medical personnel. A small share of highly trained personnel is compensated by a larger share of clinical officers (a middle-level cadre) but not by a larger share of untrained health workers. Clinical officers are relatively equally distributed. Distributional inequalities tend to be more pronounced when under-five deaths are used as an indicator of health care needs. Conversely, if health care needs are measured by HIV prevalence, the distributional inequalities appear to decline.

**Conclusion:**

The measure of inequality in the distribution of the health workforce may depend strongly on the underlying measure of health care needs. In cases of a non-uniform distribution of health care needs across geographical areas, other measures of health care needs than population levels may have to be developed in order to ensure a more meaningful measurement of distributional inequalities of the health workforce.

## Background

During the last few years, much attention has been paid to the general shortage of health workers in low-income countries, [1,2] and to the crucial importance of reducing it to attain the Millennium Development Goals [3-5]. In addition to the general shortage of health workers in these countries, there is a common understanding that large in-country inequalities exist in the distribution of health workers. So far, the evidence to support this proposition has been limited, owing to a lack of reliable disaggregated data at the country level. In this paper, we use the last census of human resources for health in Tanzania in order to describe the distributional patterns of the health workforce in the country.

Inequalities in the distribution of health workers are often described by comparing the number of health workers per capita across districts or other local administrative units [6-8]. Following this approach, the first aim of this paper will be to provide a quantitative description of inequality in the allocation of health workers per capita at the district level in Tanzania. We will show that considerable inequalities prevail across districts. While several existing studies confine themselves to the distribution of a single cadre, such as general practitioners or nurses [5,7,9], we describe the distribution both at the aggregate level and at the cadre level. In this way, we are able to study, for instance, whether districts that have relatively few physicians are "compensated" by having relatively more lower-cadre workers.

It is not obvious, though, that an equitable distribution of health workers would entail an equal number of health workers per capita across regions or districts. The need for health services per capita – and therefore the human resource requirements per capita – may vary across geographical entities due to differences in morbidity and mortality patterns. Furthermore, the composition of aggregate morbidity and mortality may differ according to area. This may have implications for health workforce planning if governments do not give equal priority to preventing and treating all conditions (e.g. by according higher priority to the health care needs of children compared to the elderly). Also, a higher number of staff per capita might be needed in areas with a lower population density.

In the literature on inequalities in the distribution of health workers in high-income countries, crude death rate has been proposed as an alternative to population as a measure of health care needs [10-12], the argument being that a high death rate is a signal of an ageing population with high health care needs.

In a low-income setting, crude death rates are probably less suitable as a measure of health care needs in the context of health workforce planning. First, due to resource constraints, governments in these countries have generally chosen to put less emphasis on the health care needs of the elderly, compared to high-income countries. Second, the elderly constitute a smaller proportion of the total population in high-fertility settings.

We therefore propose two alternative indicators of health care needs for a low-income setting: the under-five mortality rate and the HIV prevalence ratio. While both indicators clearly provide incomplete descriptions of the need for health services, they serve the purpose of drawing attention to the possibility of in-country variations in health care needs per capita that need to be taken into account when assessing the distribution of the health workforce. In the case of Tanzania, such in-country differences appear to be of sufficient significance to warrant a deviation from the principle of an equal number of health workers per capita in all districts. In practice, however, it will be necessary to come up with more comprehensive measures of need than the two partial indicators applied in this paper.

Following the economics literature on the measurement of inequality in the distribution of income, we use the Lorenz curve and the Gini index in order to characterize inequality in the distribution of health workers per capita. In addition, we present a novel way to illustrate the difference between the per capita approach (i.e. the allocation of health workers according to population) and alternative indicators of health care needs. By using concentration curves – extensively used to depict socioeconomic inequalities in health [13] – to describe alternative ways of measuring health care needs, and by drawing concentration curves in the same diagram as the Lorenz curve, we are able to illustrate graphically the significance of alternative indicators of health care needs, as well as to compare the actual distribution of health workers with the equitable distribution according to alternative measures of need. Moreover, we show how concentration curves may be usefully applied to analyse skill-mix inequalities.

The paper is organised as follows. In the following section, we present a brief introduction to the Tanzanian health system, key health indicators and the human resource situation in the health sector. This is followed by a presentation and discussion of the methods for analysing inequalities in the distribution of health workers. Data sources are presented in the subsequent section before presenting important findings. We then highlight and discuss the major issues raised in the analysis. Finally, conclusions and policy recommendations are presented at the end of the paper.

### The context

Tanzania, with 37.6 million inhabitants [14], is one of the world's poorest countries. About 36% of all Tanzanians live below the poverty line of one US dollar a day [15].

Administratively, mainland Tanzania is divided into 21 regions with 125 districts. At the district level, health services are provided through the district hospitals and the associated health centres, dispensaries and health posts. There are referral hospitals in each region. Four of these hospitals serve as tertiary hospitals for larger geographical areas.

According to the 2006 *World health report*, mainland Tanzania has a total of 48508 health workers, of whom 822 are physicians and 13292 are nurses [1]. Tanzania has the lowest physician/population ratio in the world. However, the underlying HRH data source shows that the country also has 717 Assistant Medical Officers with practical clinical skills comparable to those of physicians. In addition, there are 5642 clinical officers, who undertake a substantial share of the clinical practice [16]. Medical assistants, with little or no formal training, constitute a large share (40%) of the health workforce.

The under-five mortality rate has declined over the last decade from 147 per thousand live births in 1995–1999 to 112 in the period 2000–2005 [17]. The HIV prevalence rate is 7% [18].

## Methods

### Inequality of what?

The underlying normative idea when characterizing inequalities in the distribution of health workers is that an equitable distribution can be realized by allocating health workers according to the need for health care. To measure health care needs is not a trivial task, however. For reasons of simplicity, population levels have come to be a popular indicator of need in many practical applications, implying that inequalities in the distribution of health workers have been characterized by inequalities in the number of health workers per capita [19,20].

Population levels may not be a good measure of health care needs if disease patterns vary between locations. Some studies in developed countries have therefore proposed to replace population levels with crude death rates. For example, Gravelle & Sutton and Johnson & Wilkinson [12,21] have argued that crude deaths is a good proxy of the health care needs of a population because areas with high death rates are typically areas with an ageing population, which requires many labour-intensive health services.

As argued above, "crude deaths" may be a less suitable proxy for health care needs in low-income country settings in the context of health workforce planning. Due to the lack of alternative, comprehensive measures of health care needs, we confine our analysis to two partial measures: (1) the under-five mortality rate, and (2) the HIV/AIDS prevalence rate.

Although these measures serve mainly as illustrations here, they also capture important aspects of health care needs in a low-income setting. As many as 30% of annual deaths in low-income countries are children under the age of five, compared to less than 1% in high-income countries [22]. A large share of under-five deaths can be prevented by interventions delivered through the health system [23,24].

Moreover, in Tanzania the under-five mortality ratio varies by a factor of more than 6 between districts – from 40 deaths in Ngorongoro district to 250 deaths per 1000 live births in Ruangwa district [15]. Under-five mortality is also acknowledged by the government as one of four factors that determine the allocation of financial resources in the health sector, together with population, poverty levels and remoteness. It is therefore reasonable to use the number of under-five deaths as an indicator of health care needs, albeit a partial one.

The HIV/AIDS prevalence rate is a second possible indicator of health care needs. HIV/AIDS is imposing huge burdens on the health workforce in many low-income countries [25]. A study from Tanzania showed that the duration and frequency of hospital admission was two times higher for HIV/AIDS patients than for those with other diseases [26]. Moreover, the rapid roll-out of ART treatment is placing great demands on the health workforce [27]. HIV/AIDS is also a major cause of health worker absenteeism and attrition [28,29]. One study conducted in Tanzania [30] showed that about 26% of health workers were granted paid sick leave due to HIV/AIDS-related illnesses. Hence, a high burden of HIV/AIDS is likely to increase the need for health workers significantly. At the same time, large variations in HIV/AIDS prevalence rates have been documented in Tanzania, from 2% in Kigoma and Manyara regions to 13.5% in Mbeya region [18]. The variation in HIV/AIDS prevalence may therefore serve as one possible indicator of the variation in the need for health workers.

A natural objection to using under-five deaths, as well as other measures of the burden of disease, as a proxy for the need for health workers is that a high burden of disease may be caused by a low number of health workers [3]. If all variation in, for instance, the under-five mortality were due to unequal distribution of health workers, differences in the number of under-five deaths would not provide any reason to allocate health workers otherwise than in proportion to population. We justify our approach by showing that the number of health workers per capita can potentially explain only a small share of the variation in under-five mortality rates in Tanzania. We are not aware of any study that has argued convincingly that the number of health workers per capita is a strong predictor of HIV prevalence. (Note: Madigan et al. [31] argue that health worker density has an impact on HIV/AIDS prevalence. However, their regression analysis fails to control for variables that one would expect are important predictors of HIV/AIDS prevalence, such as sexual behaviour and attitudes, and knowledge about the transmission of the disease. Moreover, female literacy, a variable that the authors claim to be closely related to HIV/AIDS prevalence, is not included in their regression model.)

### Measuring inequality

#### Lorenz curves and the Gini index

**Figure 1:**
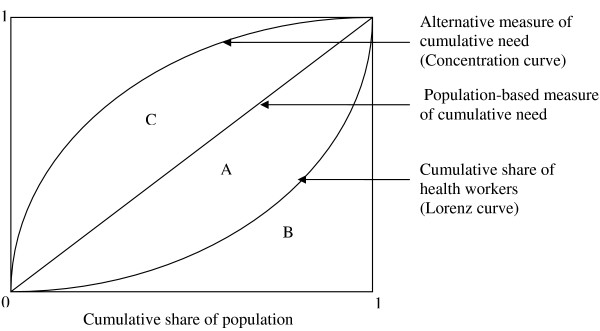
**The Lorenz curve and the concentration curves**.

We use Lorenz curves in order to characterize the distribution of health workers per capita. The Lorenz curve shows the cumulative share of health workers against the cumulative share of the population when the different locations are ranked from the lowest to the highest number of health workers per capita (see Figure 1).

We use the Gini index as a measure of the aggregate level of inequality. The Gini index takes the values between 0 and 1, with higher values indicating higher levels of inequality. Graphically, the Gini index is the area A/(A+B) in Figure 1. For discrete distributions where the observations have been ranked from below, the Gini index can be calculated as

$$G=\frac{\sum_{i=1}^{n}\left(2i-n-1\right)X_{i}}{n^{2}\mu },$$

where *G *is the Gini index, *n *is the number of observations, *X*_*i *_is the number of health workers in the i*th *location and *μ *is the mean number of health workers.

### Concentration curves and the concentration index

Concentration curves, which have been extensively used to characterize socioeconomic inequalities in health [13], are here used to characterize the need for health workers. Thus, our concentration curves plot cumulative expressions of need (i.e. the cumulative number of inhabitants, under-five deaths, and HIV+ cases) against cumulative population. In contrast to the Lorenz curve, concentration curves are constructed by ranking observations by some external variable. By using the number of health workers per capita as the external variable, we are able to superimpose the concentration curves in the same diagram as the Lorenz curve (see Figure 1). Thus, it becomes possible to make statements such as "50% of the population have access to x% of the health workers, while their need would represent y% of the aggregate need".

Obviously, if need is expressed by the number of inhabitants, the concentration curve is simply the diagonal in Figure 1. When need is expressed through other variables, the concentration curve may run both below and above the diagonal.

Concentration curves are also used in order to compare inequality in the distribution of specific cadres with inequalities in the overall distribution of health workers. We are not aware of any previous attempts to use concentration curves to characterize skill mix inequalities.

Concentration indices are calculated in order to measure whether inequalities on average are increased or reduced by replacing the number of inhabitants with alternative measures of need. Technically, the concentration index is computed in the same way as the Gini index, and graphically, the concentration index is the area C/(A+B). When the concentration curve lies above (below) the diagonal, the area 'C' is assigned a negative (positive) value.

The concentration index takes values between -1 and +1. When the index is 0, it means that the alternative measure of need does not affect the aggregate level of inequality, compared to the case when need is measured by the number of inhabitants. When the index is negative, which would be the case if the concentration curve lies everywhere above the diagonal, health care needs per capita are on average larger in the districts with the fewest health workers per capita. Hence, the inequalities are larger when we use the alternative measure of need. The opposite is true when the concentration curve lies everywhere below the diagonal, which would imply a positive concentration index.

### Data sources

Data on the number of health workers were retrieved from the Ministry of Health's Human Resources for Health census [16], the same source as was used to extract figures for the World Health Organization's Global Atlas of the Health Workforce. The HRH census encompasses all health workers in the public, private-for-profit and private not-for-profit sectors in mainland Tanzania. The data were collected at the health facility level by asking the person in charge to provide a complete list of the employees. The census is the most comprehensive and reliable source of HRH data in Tanzania at present. The HRH data may be biased due to incompleteness of the data collection process. Since we do not have any reason to believe that the degree of completeness varies systematically between districts, it is unclear how such bias might affect our results.

At the time of the census, the total number of districts was 113 (as a result of government reorganization, some districts have since been split). Following the country's official classification of districts, 22 districts are classified as urban. These consist of the regional capitals in 19 regions in addition to the three districts of Dar es Salaam region. The remaining 91 districts are classified as rural. (Note: One of the regional capitals (Babati district in Manyara region), is classified as a rural district in the Tanzanian official statistics.)

Mortality data were obtained from the National Bureau of Statistics (NBS). The data were based on the 2002 population and housing census [32] and were collected by putting questions about birth history to women of reproductive age (15–49 years). Recall bias is likely to weaken the reliability of this data source. However, more reliable reports of vital statistics are not available. Note that recall bias is not likely to affect our results insofar as there are no systematic differences in the bias across districts.

Data on HIV prevalence were based on the HIV/AIDS indicator survey of 2003–2004 [18]. These data have been estimated only at a regional level. The analysis that uses HIV prevalence data was therefore conducted at the regional level only.

## Results

### Distribution of health workers

Some health workers are employed in administrative positions in the central government. We excluded these workers from the data and remained with a total of 46 896 health workers. Their distribution across cadres and sectors is shown in Table 1.

**Table 1:** Distribution of health workers across cadres and sectors (%) (n = 46 896)

	**Government**	**Private**	**Voluntary agencies**	**Total**
Medical officer	0.8	0.3	0.2	1.3

Assistant medical officer	1.0	0.2	0.3	1.5

Clinical officer	9.0	1.1	1.7	11.7

Nurse/Nurse-Midwife	18.3	2.1	7.4	27.8

Medical attendant	30.7	1.6	7.9	40.2

Other	10.6	1.5	5.3	17.5

**Total**	**70.3**	**6.7**	**22.9**	**100.0**

On average, there are 1.4 health workers per 1000 people in Tanzania. The number of health workers per capita varies greatly between districts, from 0.3 per 1000 in Bukombe district to 12.3 per 1000 in Moshi district.

**Figure 2:**
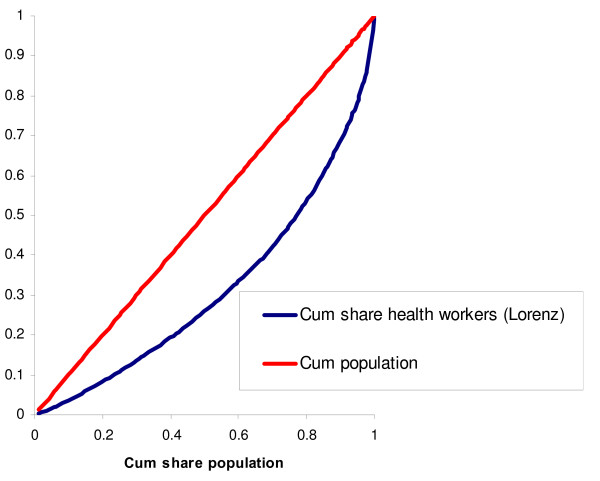
**Lorenz curve for the distribution of all health workers across districts**.

Figure 2 shows the Lorenz curve for the distribution of health workers across districts. There is significant inequality in the distribution of health workers per capita. The population quintile with the fewest health workers per capita has only 8% of the health workers, while the quintile with the most health workers has 46% of the workers. The value of the Gini index is 0.229.

Part of the inequality in the distribution of health workers is driven by an urban/rural divide. Urban districts have on average more than twice as many health workers per capita as rural districts (see Table 2). Seventeen of the 22 urban districts are among the top 20 districts, ranked by the number of health workers per capita. It is true that there are some urban districts with very few health workers per capita, but these districts are located in Dar es Salaam not far from the national hospital, which happens to be located in a different district.

**Table 2:** Urban/rural distribution of health workers

	**Health workers per 1000**			**Gini index**
		
	Average	Minimum	Maximum	
Urban districts	3.0	0.6	12.3	0.225

Rural districts	1.1	0.3	3.0	0.110

All districts	1.4	0.3	12.3	0.229

We calculated the Gini index for urban and rural districts separately and found that the Gini index for the urban subsample was almost as high as for the country as a whole (0.225). Hence, significant inequalities exist across urban districts, even though their average number of health workers is much higher than in rural districts. In the rural subsample, on the other hand, the inequalities between districts are much smaller. The Gini index is only 0.11. The most significant inequalities are thus the inequalities between rural and urban districts and among urban districts.

### Skill mix

**Figure 3:**
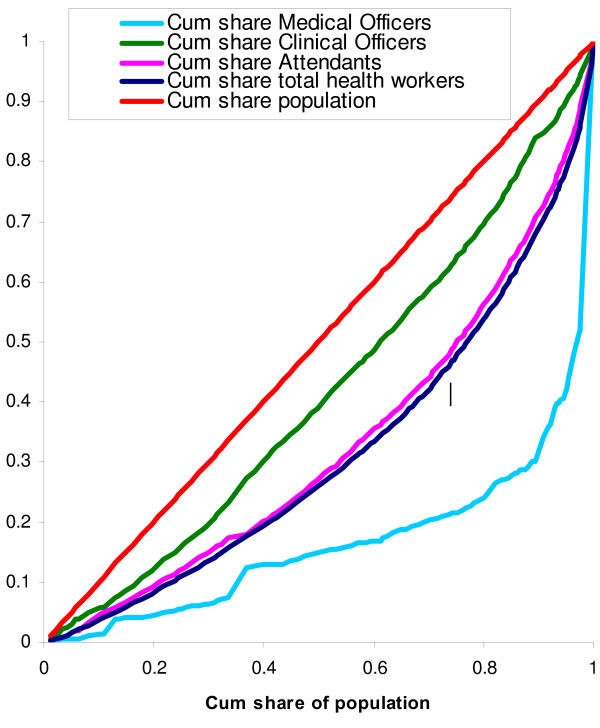
**Distribution of health workers per capita by cadre in all districts**.

Some cadres are more unequally distributed than others across districts. Figure 2 shows the Lorenz curve for the cumulative share of all health workers, together with the concentration curves for selected cadres. Cadres not displayed in Fig. 3, such as assistant medical officers and nurses, were distributed quite similarly to the aggregate health workforce.

Those districts that have a small share of the health workforce (relative to their population level) have an even smaller share of the highly trained medical personnel (medical officers and specialists). The concentration curve for this group lies everywhere below the Lorenz curve and the concentration index is as high as 0.595.

How do the disadvantaged districts compensate for their small share of highly skilled health workers? Interestingly, medical attendants, who have little or no training, do not constitute a larger share of the workforce in these districts compared to the more advantaged ones. Indeed, the concentration index for the medical attendants is 0.195, which is very close to the Gini coefficient. Indeed, the concentration curve shows that medical attendants are distributed quite similarly to the distribution of the total health workforce.

The skill mix in the disadvantaged districts is characterized, however, by a relatively large share of clinical officers. The concentration index for clinical officers is only 0.006, suggesting that clinical officers are distributed quite equally according to population levels.

Hence, the skill mix in the disadvantaged districts is marked by few highly trained people but relatively more health workers with medium-level skills. But there is no cadre of which the disadvantaged districts have a larger share of the health workers than is suggested by their relative population levels (i.e. all concentration curves in Fig. 3 fall below the diagonal).

### Alternative measures of need

**Figure 4:**
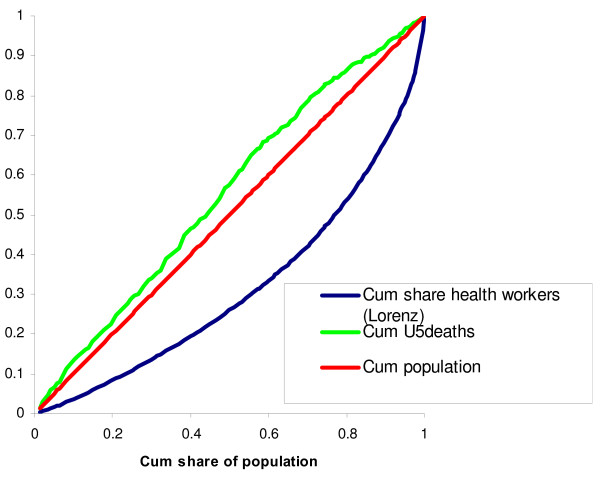
**Cumulative share of total health workers and U5 deaths across districts**.

One alternative to population levels as a measure of need is the number of under-five deaths. In Fig. 4, the concentration curve for the cumulative share of under-five deaths is shown together with the Lorenz curve for the cumulative share of all health workers. The concentration curve for under-five deaths lies everywhere above the diagonal, showing that those districts that have few health workers per capita at the same time have a large share of under-five deaths per capita (the concentration index is -0.26 for all districts, -0.29 for urban districts and -0.22 for rural districts, respectively). In other words, the need for health services – measured as the number of under-five deaths – in districts with few health workers is larger than suggested by their respective population levels.

**Figure 5:**
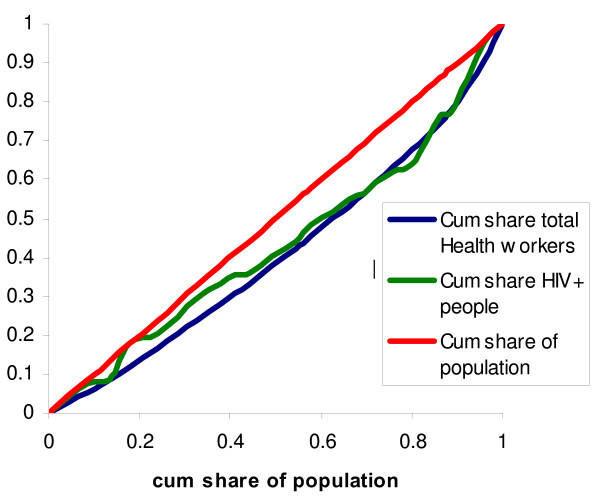
**Distribution of total health workers and HIV prevalence in 21 regions**.

A second alternative measure of need is the HIV prevalence rate. Unfortunately, these data are available only at the regional level. Figure 5 shows the regional-level Lorenz curve for the cumulative share of health workers, together with the concentration curve for the cumulative share of HIV-positive persons.

Interestingly, this measure of need shows a remarkably different pattern than that for under-five deaths. The concentration index is 0.077, which is not very different from the regional level Gini index of 0.117. This implies that at the regional level, health workers are on average distributed fairly well according to need as measured by the HIV prevalence rate. However, the concentration curve also shows that there are individual regions where the number of health workers does not correspond at all to the number of HIV-infected persons.

Table 3 reports part of the data material behind Figs. 2, 3, 4, 5, comparing the actual distribution of health workers with alternative measures of need for each population quintile.

**Table 3:** Distribution of health workers relative to population and alternative indicators of health care needs

**District level data (113 districts)**		
Measures of need		

Share of population	Share of U5 deaths	Share of health workers

20%	28%	8%

40%	50%	19%

60%	70%	33%

80%	87%	54%

	Concentration index = -0.266	Gini index = 0.229

**Regional level data (21 regions)**		

Measures of need		Share of health workers

Share of population	Share of HIV+ people	

20%	20%	16%

40%	37%	30%

60%	53%	50%

80%	65%	70%

	Concentration index = 0.077	Gini index = 0.118

## Discussion

This study is a first attempt to describe and measure systematically the level of inequality in the distribution of the health workforce in Tanzania, using the Lorenz curve and the Gini index as well as concentration curves and indices. It is also a first attempt to use alternatives to population levels as proxy indicators of health care needs when measuring distributional inequalities of the health workforce in a low-income setting.

Our findings indicate that there are large inequalities in the number of health workers per capita across districts, with a 40-fold difference between districts at the high end of the distribution compared to the district at the lower end. Of course, some of these differences are planned for. The referral system implies that some districts are supposed to serve populations from other districts through the regional and tertiary hospitals. As a consequence, we would expect a higher concentration of health workers relative to the population in districts hosting a referral hospital. One way of addressing this problem would be to exclude regional and tertiary referral hospitals from the analysis. Doing so, the results reported in Table 2 would change and appear as in Table 4.

**Table 4:** Urban/rural distribution of health workers (excluding regional and tertiary hospitals)

	**Health workers per 1,000**			**Gini index**
	Average	Minimum	Maximum	

Urban districts	1.4	0.6	3.2	-

Rural districts	1.1	0.3	3.0	-

All districts	1.1	0.3	3.2	0.070

As expected, the number of health workers per capita in the urban districts drops dramatically. Still, however, urban districts have almost 30% more health workers per capita compared to the rural districts. However, this estimate of the inequality is likely to be biased strongly downward, because regional hospitals also serve as district hospitals in their respective locations. An unbiased analysis would therefore exclude only those workers at these hospitals who are needed for their regional referral services, and not all workers, as we have done above.

More importantly, Table 4 shows that even after excluding the regional and tertiary hospitals, there is a tenfold difference in the number of health workers per capita between districts at the high end of the distribution compared to the district at the lower end.

Our results also point to huge differences between urban districts in their availability of health personnel (0.6–12.3 health workers per 1000 people). However, part of this difference could be attributed to the fact that only a few urban districts host tertiary hospitals. We therefore recalculated our results excluding the tertiary referral hospitals. The inequalities are then reduced, but there is still more than a fivefold difference (0.6–3.2) between the urban districts with the lowest and highest number of health workers per capita. The Gini index is reduced from 0.225 to 0.081.

As previously noted, part of the inequalities between urban districts can be explained by the fact that two of the three districts in Dar es Salaam have few health workers, while their populations are partly served by the national hospital located in the third district. This observation points at a more fundamental problem in the way inequalities are measured both in this and in other studies: service provision does not always follow district boundaries. One author [33] has succinctly argued that "the geographical areas that are implicit in any population to physician ratio present two major problems. First, the geographical areas tend to be artificial and do not necessarily reflect the natural geographical pattern of health care delivery and consumption...Secondly, and somewhat related to the first point, is the assumption that all health care consumption and delivery activities take place within the defined geographic area. Such an assumption is often untenable". It is not unreasonable to assume that those places that have more health workers per capita will to some extent attract patients from neighbouring districts, due to a perceived higher quality of service. With such crossovers, it may be argued that the standard way of estimating health worker inequalities will bias the estimates upwards.

Unfortunately, our data set does not allow us to study in-district differences in the distribution of the health workforce. Many Tanzanian districts are relatively large (the mean size of a rural district is around 9000 km^2^), and differences within districts may be larger than differences between districts. There is reason to believe there may be large differences in the number of health workers per capita between the remote and the more central parts of each district. Hence, this study may underestimate the true differences in the distribution of the health workforce.

### Skill mix and quality of services

By disaggregating the health worker distribution by cadre, we were able to study the skill-mix distribution between districts. The use of concentration curves for the distribution of each cadre in combination with the Lorenz curve for the distribution of the total health workforce illustrates a new way of analysing the relationship between inequalities in the total health workforce and the skill mix.

Differences in the skill mix may cause differences in the quality of the health workforce, which in turn may affect the quality of health services. There is a concern that the most disadvantaged districts not only have the lowest number of health workers per capita but also a disproportionately large share of the less-well-trained workers and therefore an even poorer access to quality health services than suggested by the aggregate number of health workers.

Our results confirm that districts with few health workers per capita also have a disproportionately small share of highly trained health workers. Hence, the inequality in access to health services of good quality is likely to be even larger than suggested by the inequality in the distribution of the total health workforce.

### Alternative measures of need

Due to the variation across districts in the disease patterns, we suggested reanalysing the distribution of the health workforce by using alternatives to the standard measure of health care needs (i.e. the level of population). By combining the use of concentration curves for these alternative measures of need with the Lorenz curve of the actual distribution of health workers, this paper suggests a novel and illuminating way to compare the implications of alternative measures of need.

The two alternatives considered – the share of under-five deaths and the share of HIV-infected persons – both clearly deviate from the standard measure of need. The implications for the degree of inequality differ, however, depending on which alternative measure is used. Under-five deaths are more highly concentrated in areas with a relatively small share of the health workforce, and inequality in the distribution of the health workforce will therefore become more pronounced by using this measure of need, compared with the standard measure. HIV, on the other hand, is more concentrated in urban areas where the supply of health workers is more abundant, suggesting that this measure of need will cause a reduction in the implied inequalities in the distribution of health workers. Our results suggest that much relevant information may be left out when population is used as the only measure of need, i.e. when distributional inequalities are described solely by differences in the number of health workers per capita. One way to capture this information would be to build more comprehensive measures of health care needs than we have been able to do in this paper, by measuring differences in the disease burden across different parts of the country and how these differences translate into health care needs.

### Policy implications

The major criterion for allocating health workers across districts in Tanzania is relative population levels. The observation that health care needs may differ substantially between districts in Tanzania might suggest that other factors should be considered as well. Like the financial allocation formula used by the Ministry of Health [34], which combines the levels of population with other indicators of need, additional factors might be built into the allocation formula for a more sensible and fairer distribution of the health workforce.

One possible argument against the appropriateness of using alternative needs-based allocation formulas is that there may be a causal relationship between the number of health workers and the observed need for health care. In the extreme, if all variation in disease burden were caused by differences in the number of health workers, there would be no reason to deviate from the standard allocation rule (i.e. population levels). In reality, however, there are many other factors that might explain the differences in disease burden. With regard to the alternative measures of need used in this paper, there is no indication that differences in the number of health workers per capita can explain the observed differences in the HIV prevalence rates, because there are more HIV cases in those places where there are many health workers.

When it comes to under-five deaths, on the other hand, Anand and Bärnighausen [3] have argued that a low number of health workers per capita causes increased under-five mortality (in a cross-country data set). Multivariate regression analysis on the Tanzanian data set shows, however, that health worker density can potentially explain only a small share of the variation in under-five deaths across districts in Tanzania. We regressed the number of under-five deaths per capita against the number of health workers per capita, using four different groups of health workers. The linear model was able to explain only 12.5% of the total variation in the dependent variable, while a non-linear model including also the squared variables explained 19.9% of the variation (see Table 5). This suggests that factors other than health worker density explain the major share of the variation in under-five deaths in Tanzania. Hence, we conclude that there is a case for using under-five mortality, along with other indicators of need, in the allocation of the health workforce.

**Table 5:** Relationship between health worker density and under-five mortality

**Dependent variable**	**R**^2^	**Independent variables**	**Coefficient**	**Standard error**	**P-value**
*Under-five deaths and health worker density. Linear model*					

Under-five deaths per capita	0.125	Medical officers/capita (MO)	4.56	4.57	0.321

		Clinical officers/capita (CO)	-3.28	2.17	0.134

		AMOs and others/capita (AMO+)	-0.47	0.46	0.301

		Attendants/capita (ATT)	0.04	0.61	0.942

*Under-five deaths and health worker density. Non-linear model*					

Under-five deaths per capita	0.199	MO	20.38	13.59	0.137

		CO	-9.43	5.87	0.111

		AMO+	-2.04	0.82	0.015

		ATT	1.08	1.24	0.383

		MO2	-32658.62	28416.52	0.253

		CO2	17247.5	11915.73	0.151

		AMO+2	226.57	146.67	0.125

		ATT2	-372.43	458.08	0.418

Of course, if health care needs are systematically higher in areas with low health worker densities, it will make sense to use a population-based allocation of health workers as a first step, before further refining the allocation formula. However, as shown in our analysis of the distribution of HIV prevalence, it is possible that health care needs are higher in areas where health worker densities are also high. Therefore, a comprehensive analysis of health care needs seems appropriate in designing well-targeted policies to reduce distributional inequalities.

One weakness of the analysis is our inability to conduct district-level analysis using HIV prevalence as an indicator of health care needs, which is because the data are disaggregated only down to the regional level. Our results therefore do not produce strong policy implications for how HIV prevalence can be taken into account in the actual allocation of health workers to districts in Tanzania. (The high p-values should not be taken to imply that there is no relationship between the number of health workers per capita and the number of under-five deaths per capita. Large confidence intervals may be due to high correlation between the independent variables.)

## Conclusion

Superimposing concentration curves for health care needs in the same diagram as the Lorenz curves for the distribution of the health workforce provides a simple and clear graphical illustration of the importance of alternative indicators of health care needs for the measurement of health worker distributional inequalities. Moreover, superimposing concentration curves for the cadre-specific distribution provides an illuminating way to analyse the relationships between distributional inequalities in the total health workforce and skill mix inequalities. A proper understanding of the skill mix inequalities is, in turn, fundamental for understanding differences in access to good-quality health services between populations in worse-off and better-off districts.

The study acknowledges the usefulness of population levels as an indicator of health care needs and thus as a basis for measuring distributional inequalities in the health workforce. But in settings where the disease burden is not uniformly distributed, relying solely on population as a measure of health care needs may lead to the omission of much relevant information necessary for the accurate measurement of need, and consequently for a more sensitive distribution of health personnel relative to need. One way of capturing this information would be to identify and apply more comprehensive measures of health care needs than we have been able to do in this paper. To do this, more research is needed to identify more sensible indicators for the measurement of health care needs and on how to "weigh" the identified indicators together into one composite measure. This requires multidisciplinary teamwork involving economists, epidemiologists and human resource planning specialists.

## Competing interests

The authors declare that they have no competing interests.

## Authors' contributions

MAM and OM equally participated in designing the study, analysing the data and drafting all sections of the manuscript. Both authors have read and agreed to the paper being submitted as it is.
